# Three in every four systematic reviews and meta‐analyses on concomitant anterior cruciate ligament reconstruction and anterolateral complex procedures present at least one type of spin in the abstract

**DOI:** 10.1002/ksa.12787

**Published:** 2025-07-13

**Authors:** Adela Gottardi, Kieran Chalmers, Prushoth Vivekanantha, Jeffrey Kay, Mikael Sansone, Darren de SA

**Affiliations:** ^1^ Schulich School of Medicine and Dentistry Western University London Ontario Canada; ^2^ Michael DeGroote School of Medicine McMaster University Hamilton Ontario Canada; ^3^ Department of Surgery, Division of Orthopaedic Surgery McMaster University Hamilton Ontario Canada; ^4^ University Hospital University of Gothenburg Gothenburg Sweden

**Keywords:** bias, lateral extra‐articular tenodesis, meta‐analysis, spin, systematic review

## Abstract

**Purpose:**

Spin is a form of bias that misrepresents research findings, potentially influencing clinical decisions and patient care. This meta‐research study evaluates the prevalence and types of spin in abstracts of systematic reviews and meta‐analyses comparing anterior cruciate ligament reconstruction (ACLR) with and without lateral extra‐articular tenodesis (LET) and anterolateral ligament reconstruction (ALLR). A secondary aim is to assess review quality per AMSTAR‐2 criteria and associations between spin and review characteristics.

**Methods:**

A systematic search of PubMed, EMBASE and MEDLINE[Ovid] in May 2024 identified eligible reviews. Two reviewers independently assessed the nine most severe types of spin and methodological quality using AMSTAR‐2. Review characteristics, including publication year, total citations, average yearly citations and journal impact factor, were analyzed for associations with spin.

**Results:**

Of 24 included reviews, 75% (18/24) contained at least one form of spin, with Type 3 spin, ‘Selective reporting or overemphasis on efficacy outcomes favoring the experimental intervention’, being the most common (62.5%, 15/24). Reviews with spin had a significantly more recent median publication year (*p* = 0.011), while those without spin had significantly higher total citation counts (*p* = 0.021). No significant differences were observed in average yearly citations or impact factors between groups. Reviews with three or more types of spin were published more recently than those with fewer (*p* = 0.007), with no significant differences in total citations, average yearly citations or impact factor. Most reviews (91.7%) were rated as critically low‐quality using AMSTAR‐2, with substantial inter‐reviewer agreement (*κ* = 0.839).

**Conclusion:**

Spin is highly prevalent in systematic reviews and meta‐analyses evaluating ACLR with and without LET or ALLR, particularly in recent publications. Given that many reviews exhibit critically low methodological quality, efforts should focus on strengthening adherence to reporting standards and reducing spin to ensure the publication of unbiased evidence that informs clinical practice.

**Level of Evidence:**

Level IV.

**Abbreviations**
ACLR: anterior cruciate ligament reconstruction
ALLR: anterolateral ligament reconstruction
AMSTAR‐2: revised assessment of multiple systematic reviews 2
IKDC: International Knee Documentation Committee
LET: lateral extra‐articular tenodesis
MA: meta‐analysis
PRISMA: Preferred Reporting Items for Systematic Reviews and Meta‐Analyses
RCT: randomized controlled trials
RoB: risk of bias
SR: systematic review

## INTRODUCTION

Anterior cruciate ligament (ACL) tears are common knee injuries with significant implications for patient mobility, athletic performance [22], and quality of life [16], with an incidence around 68.6 per 100,000 person years [43]. ACL reconstruction (ACLR) is the standard treatment, which has been increasing in frequency [53] and utilizes a variety of graft options and techniques [37]. Despite the success of isolated ACLR, residual rotational instability and/or ACL re‐injury remains a challenge in young (under 25 years), highly active patients, with risk factors for re‐tear such as hypermobility [17]. Anterolateral complex procedures, such as lateral extra‐articular tenodesis (LET) and anterolateral ligament reconstruction (ALLR), have been introduced as adjuncts to ACLR to improve functional outcomes and lower rates of re‐rupture [13, 41]. The rapidly expanding body of literature on anterolateral complex procedures highlights its increasing adoption and ongoing debate regarding its role in optimizing ACLR outcomes.

Systematic reviews (SRs) and meta‐analyses (MAs) are critical in evidence‐based medicine, synthesizing data to assist with clinical decision‐making [44]. They play an important role in presenting the best available evidence on a topic, particularly new and evolving topics such as comparing ACLR with or without concomitant LET or ALLR and highlighting common themes or conflicting results. Despite their value, these reviews are not immune to bias, which can distort findings and misinform clinical practice.

‘Spin’ is a specific type of bias that involves the misrepresentation of data or study results. This bias is defined as ‘a specific way of reporting, intentional or not, to highlight that the beneficial effect of the experimental treatment, in terms of efficacy or safety, is greater than that shown by the results’ [51]. A previously established framework for identifying the nine most severe types of spin provides a foundation for evaluating its prevalence in the abstracts of SR and MA. Key themes that are present in spin include, but are not limited to, emphasizing secondary outcomes over primary ones, focusing on statistically significant findings while ignoring unfavourable results, or failing to acknowledge limitations and methodological flaws. This issue is particularly concerning in the abstracts of SRs and MAs, which often serve as the basis for clinical decision‐making, particularly in rapidly evolving fields such as sports medicine and orthopaedic surgery. Prior studies have demonstrated a high prevalence of spin in related sports medicine literature, highlighting the need to critically evaluate research in this area and to better understand the quality and reliability of the current orthopaedic evidence base [19, 25, 49]. Identifying and addressing spin is crucial for ensuring the integrity of evidence‐based recommendations [51].

The primary objective of this meta‐research study is to assess the prevalence and types of spin in the abstracts of SR and MA in ACLR with concomitant LET or ALLR compared to isolated ACLR. A secondary objective is to investigate whether spin correlates with review characteristics, including year of publication, number of citations, journal impact factor and AMSTAR‐2 score. By addressing these questions, this study aims to promote transparency and improve the quality of evidence and reporting guiding ACLR treatment strategies.

## METHODS

### Search criteria

PubMed, EMBASE and MEDLINE[Ovid] were searched from database inception until 22 May 2024. The following search terms were used: ‘lateral extra‐articular tenodesis’ or ‘LET’ or ‘anterolateral ligament’ or ‘ALL’ or ‘anterolateral complex’ and ‘anterior cruciate ligament’ or ‘ACL’ (Table S1). Inclusion criteria encompassed (1) SRs and MAs published in English that compared ACLR with concomitant LET or ALLR compared to isolated ACLR and (2) evaluation of at least one clinical outcome in the form of patient‐reported outcome measures (PROMs), complications (e.g., revision rates), or objective physical examination tests (e.g., anterior tibial translation, postoperative Lachman, postoperative pivot‐shift). Exclusion criteria encompassed reviews that focused on ACL repair rather than reconstruction, those limited to biomechanical outcomes without reporting clinical outcomes, and reviews that included non‐human or non‐living human studies.

### Screening

Two independent reviewers (A.G. and K.C.) screened studies for eligibility in both the title and abstract and full‐text stages, with conflicts resolved through consensus or through consultation with a more senior reviewer (P.V.).

### Assessment of agreement

The inter‐reviewer agreement was evaluated using Cohen's kappa (*κ*) coefficient statistic for screening. *A priori* classification was determined using the following criteria: *κ* of 0.91–0.99 was considered to be almost perfect agreement; *κ* of 0.71–0.90 was considered to be substantial agreement; *κ* of 0.61–0.70 was considered to be high agreement; *κ* of 0.41–0.60 was considered to be moderate agreement; *κ* of 0.21–0.40 was considered to be fair agreement; and a *κ* value of 0.20 or less was considered to be no agreement [33].

### Quality assessment and evaluation of spin

Two authors (A.G. and K.C.) independently assessed abstracts for the nine most severe types of spin (Table 1) and independently assessed the quality of the reviews using the AMSTAR‐2 tool, with conflicts resolved through consensus or by a third author (P.V.) [29, 51]. Our evaluation focused on the nine most severe spin types, as defined in the original publication [51], to align with prior orthopaedic studies [10, 19] and to emphasize those most likely to mislead readers. To assess the methodological rigour of the 24 reviews included in our analysis, we applied the AMSTAR‐2 tool, a 16‐item checklist for evaluating SRs. This tool categorizes reviews into one of four quality levels: critically low, low, moderate or high. Each author was trained to evaluate spin and apply the AMSTAR‐2 grading by individually reviewing the original studies describing these tools.

**Table 1:** Nine most severe types of spin, as per Yavchitz et al., and the proportion of reviews containing specific types of spin after conflict resolution.

Type of spin	Description of spin	Percentage of abstracts containing spin, post‐conflict resolution
1	Conclusion contains recommendations for clinical practice not supported by the findings	33.3% (9/24)
2	Title claims or suggests a beneficial effect of the experimental intervention not supported by the findings	16.7% (4/24)
3	Selective reporting of or overemphasis on efficacy outcomes or analysis favouring the beneficial effect of the experimental intervention	62.5% (15/24)
4	Conclusion claims safety based on non‐statistically significant results with a wide confidence interval	8.33% (2/24)
5	Conclusion claims the beneficial effect of the experimental treatment despite high risk of bias in the primary studies	14.3% (3/21)
6	Selective reporting of or overemphasis on harm outcomes or analysis favouring the safety of the experimental intervention	0.0% (0/24)
7	Conclusion extrapolates the review's findings to a different intervention	0.0% (0/24)
8	Conclusion extrapolates the review's findings from a surrogate marker or a specific outcome to the global improvement of the disease	20.8% (5/24)
9	Conclusion claims the beneficial effect of the experimental treatment despite reporting bias	4.17% (1/24)

Data extraction included the year and country of publication, type of review, number of included studies, total number of patients, funding source, journal of publication and whether the review explicitly reported adherence to Preferred Reporting Items for Systematic Reviews and Meta‐Analyses (PRISMA) guidelines. Citation metrics were recorded as the total number of citations on Google Scholar as of 1 January 2025, and average yearly citations were defined as the total citations divided by the number of years from publication to 2025. Journal impact factor was retrieved from the journal's website or, if unavailable, from secondary academic databases as of 1 January 2025. We also recorded whether the review included only randomized controlled trials (RCTs) and the risk of bias (RoB) assessment method used.

### Statistical analysis

Statistics were performed using Microsoft Excel, and statistical significance was set at *p* < 0.05. The primary outcomes included the prevalence of each type of abstract spin and the AMSTAR‐2 ratings for reviewer 1, reviewer 2 and post‐conflict resolution. Inter‐rater reliability, standard error and 95% confidence intervals for the AMSTAR‐2 ratings were calculated using GraphPad Prism 10.0. AMSTAR‐2 ratings and the presence of spin were recorded as counts and percentages. Descriptive statistics, including ranges, medians and interquartile ranges (IQR), were used to analyze review characteristics. Medians (IQR) of review characteristics, including publication year, total number of citations as of 1 January 2025, number of yearly citations from the year of publication to 2025 and journal impact factor, were compared between spin and no‐spin groups using the non‐parametric Mann–Whitney *U*‐test. This same analysis was used for reviews with fewer than three types of spin and reviews with three or more types of spin. This non‐parametric test was chosen as the outcomes were non‐normally distributed as per the Shapiro–Wilk test. Graphical representations in the form of bar charts were created depicting the presence of spin based on year of publication and AMSTAR‐2 rating. Box plots were made by comparing the number of citations (total and yearly) and publication year for reviews with no spin and spin, as well as less than or greater than and equal to three types of spin.

## RESULTS

### Literature search

**Figure 1:**
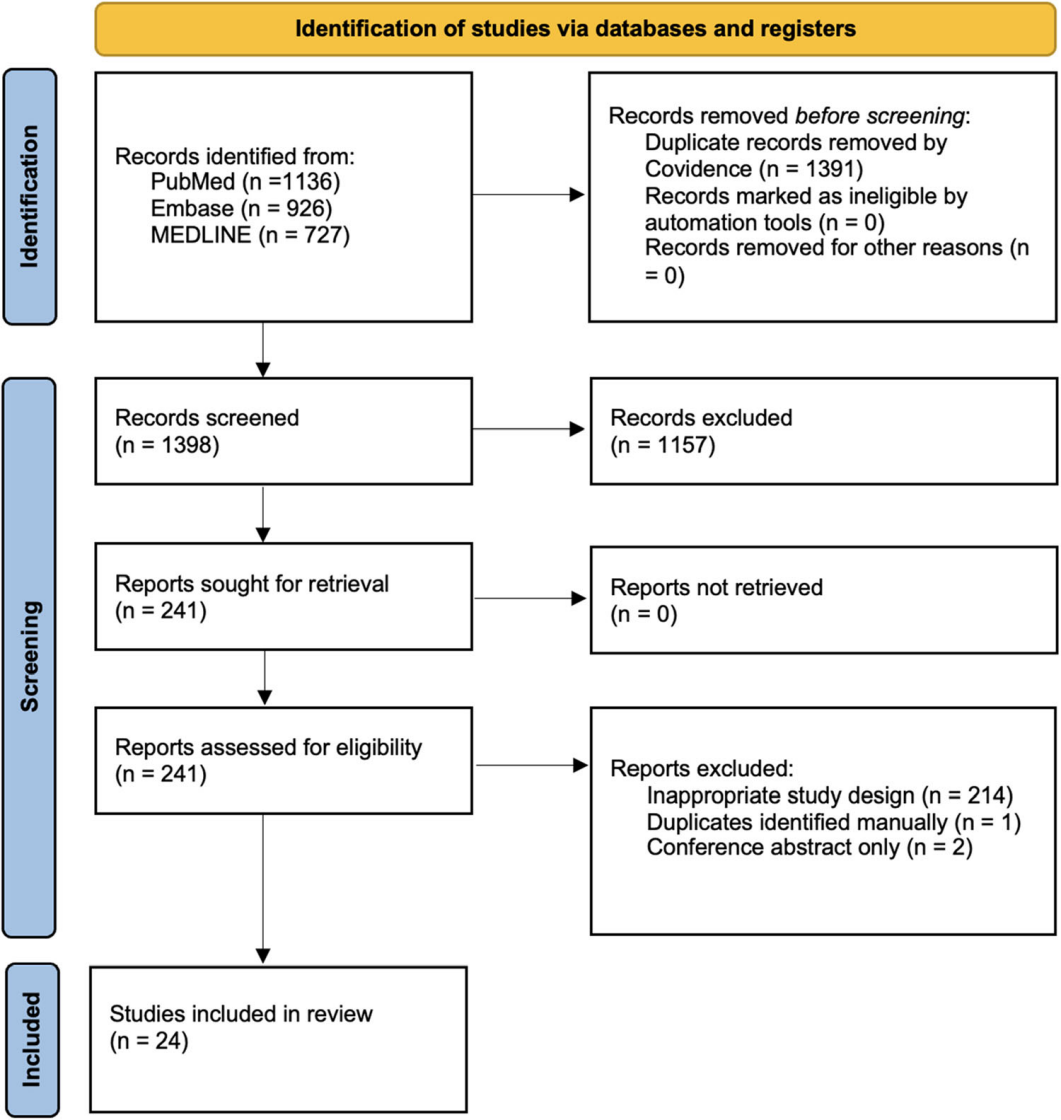
Flow diagram representing a systematic review comparing studies of anterior cruciate ligament reconstruction with or without lateral extra‐articular tenodesis or anterolateral ligament reconstruction.

The initial screening identified 2789 potentially eligible studies, with 1391 being identified as duplicates. Of 1398 remaining studies, 241 were included after title and abstract screening. After the full‐text screening, 24 reviews met the inclusion criteria and were included in the final analysis (Figure 1) [1, 2, 4, 5, 7, 9, 11, 12, 13, 15, 18, 23, 24, 30, 31, 32, 34, 35, 36, 40, 42, 47, 50, 52]. Kappa scores were 0.71 for title and abstract screening and 0.80 for full‐text screening, indicating substantial agreement.

### Review study characteristics

The included reviews were published between 2015 and 2024, with 83.3% (20/24) published in 2020 or later (Table 2). The majority (79.2%, 19/24) of the reviews were SR and MA, followed by SR without MA (16.7%, 4/24) and one SR with network MA (4.2%, 1/24). The most common countries of publication were China (20.8%, 5/24) and the United States (12.5%, 3/24). The number of studies included in each review ranged from 5 to 46 (median: 9). Total number of citations ranged from 0 to 270 (median: 26.5), and the average number of yearly citations ranged from 0 to 27 (median: 6.9). The number of patients ranged from 326 to 4956 (median: 904), with 8.3% (2/24) of reviews not reporting this value. Only 20.8% (5/24) reviews included randomized controlled trials exclusively. Regarding funding, 41.7% (10/24) received external funding, 29.2% (7/24) reported no funding and 29.2% (7/24) did not disclose funding status. The most common journals were *Arthroscopy* (29.2%, 7/24), followed by *The American Journal of Sports Medicine* (20.8%, 5/24). Impact factors ranged from 0.2 to 4.4 (median: 3.7). Most reviews (91.7%, 22/24) reported adherence to PRISMA guidelines. The Cochrane RoB tool was the most commonly used method (45.8%, 11/24) to assess RoB, while 12.5% (3/24) of reviews did not perform a RoB assessment.

**Table 2:** Summary of review characteristics, including author, review type (systematic review [SR] or meta‐analysis [MA]), year and country of publication, number of included studies, total number of included patients, funding source, total citations, average yearly citations, journal of publication, journal impact factor, PRISMA adherence, whether only randomized controlled trials (RCTs) were included, and method of risk of bias (ROB) assessment.

Author	Year of publication	Type of review	Country of publication	Number of included studies	Number of patients included in review	Funding source	Number of total citations	Average number of yearly citations	Journal	Impact factor	Prisma adherence	Included only RCTs	Method of ROB assessment
Agarwal et al. [1]	2022	SR and MA	United Kingdom	24	961	No external funding	10	3.3	*World Journal of Orthopaedics*	2	Yes	No	Critical Appraisal Skills Programme
Hurley et al. [24]	2020	SR and MA	United States	6	729	No external funding	33	6.6	*Knee Surgery, Sports Traumatology, Arthroscopy*	3.3	Yes	No	Newcastle–Ottawa Scale
Onggo et al. [35]	2021	SR and MA	Australia	7	1106	Undeclared	41	10.3	*The American Journal of Sports Medicine*	4.2	Yes	Yes	Cochrane ROB Tool
Xu et al. [50]	2020	SR and MA	China	6	828	External funding received	26	5.2	*The Journal of Arthroscopic and Related Surgery*	4.4	Yes	No	Cochrane ROB tool (RCTs); Newcastle–Ottawa (cohorts)
Na et al. [34]	2021	SR and MA	Korea	20	2376	Undeclared	60	15	*Orthopaedic Journal of Sports Medicine*	2.4	Yes	No	Cochrane ROB tool and Jadad (RCTs), Newcastle‐Ottawa (non‐RCTs)
Damayanthi et al. [11]	2024	SR and MA	Indonesia	5	797	No external funding	0	0	*Revista Brasileira de Ortopedia*	0.37	Yes	No	Joanna Briggs Institute tools
Bosco et al. [7]	2024	SR and MA	Italy	14	1830	External funding received	17	17	*The American Journal of Sports Medicine*	4.2	Yes	Yes	Cochrane ROB tool
Hewison et al. [23]	2015	SR and MA	Canada	29	4956	External funding received	270	27	*The Journal of Arthroscopic and Related Surgery*	4.4	Yes	No	Cochrane ROB tool (RCTs) and an informal modified Cochrane style assessment (non‐RCTs)
Boksh et al. [5]	2024	SR and MA	United Kingdom	10	792	Undeclared	24	24	*The American Journal of Sports Medicine*	4.2	Yes	No	Risk of Bias in Non‐Randomized Studies of Interventions tool
Ariel de Lima et al. [2]	2021	SR and MA	Brazil	20	1495	No external funding	48	12	*Knee Surgery & Related Research*	4.1	Yes	No	ROS not assessed for RCTs, Newcastle–Ottawa Scale (non‐RCTs)
Mao et al. [32]	2021	SR and MA	China	6	1010	Undeclared	16	4	*Orthopaedic Journal of Sports Medicine*	2.4	Yes	Yes	Cochrane ROB tool
Park et al. [36]	2022	SR and network MA	South Korea	11	1077	External funding received	28	9.3	*The Journal of Arthroscopic and Related Surgery*	4.4	Yes	Yes	Cochrane ROB tool
Kunze et al. [30]	2021	SR and MA	United States	46	Total not reported	External funding received	46	11.5	*The Journal of Arthroscopic and Related Surgery*	4.4	Yes	In SR – no; in MA – yes	Jadad Scale (RCTs)
Yin et al. [52]	2021	SR and MA	China	6	460	External funding received	15	3.8	*Journal of Orthopaedic Surgery*	1.3	Yes	No	Cochrane ROB tool (RCTs), NOS (non‐RCTs)
Saithna et al. [42]	2022	SR	Italy, France, United States	8	716	External funding received	19	6.3	*The Journal of Arthroscopic and Related Surgery*	4.4	Yes	No	No RCTs included, methodological Index for Non‐Randomized Studies tool (non‐RCTs)
Bucar et al. [9]	2021	SR and MA	Brazil	6	776	Undeclared	4	1	*Revista Brasileira de Ortopedia*	0.37	Yes	Yes	Cochrane ROB tool
Song et al. [47]	2015	SR	China	7	326	Undeclared	134	13.4	*The Journal of Arthroscopic and Related Surgery*	4.4	No	No	ROB not assessed
Delaloye et al. [12]	2018	SR	France	5	1691	External funding received	27	3.86	*Techniques in Orthopaedics*	0.2	No	No	ROB not assessed
Feng et al. [15]	2022	SR and MA	China	11	1745	External funding received	9	3	*Journal of Orthopaedic Surgery*	1.3	Yes	No	Cochrane ROB tool and the modified Downs & Black
Beckers et al. [4]	2021	SR and MA	Canada	11	1892	No external funding	27	6.8	*Journal of Experimental Orthopaedics*	2	Yes	No	Cochrane ROB tool (RCTs), Newcastle–Ottawa Scale (non‐RCTs)
Devitt et al. [13]	2017	SR and MA	Australia	11	847	Undeclared	87	10.9	*Orthopaedic Journal of Sports Medicine*	2.4	Yes	No	Modified Downs & Black
Grassi et al. [18]	2024	SR and MA	Italy	8	676	No external funding	7	7	*The American Journal of Sports Medicine*	4.2	Yes	No	No RCTs included; Newcastle–Ottawa Scale (non‐RCTs)
Littlefield et al. [31]	2020	SR	United States	40	Total not reported	External funding received	45	9	*The Journal of Arthroscopic and Related Surgery*	4.4	Yes	No	ROB not assessed
Rhatomy et al. [40]	2022	SR and MA	Indonesia	8	683	No external funding	20	6.7	*European Journal of Orthopaedic Surgery & Traumatology*	1.4	Yes	No	Coleman methodology score

### Review study quality

**Figure 2:**
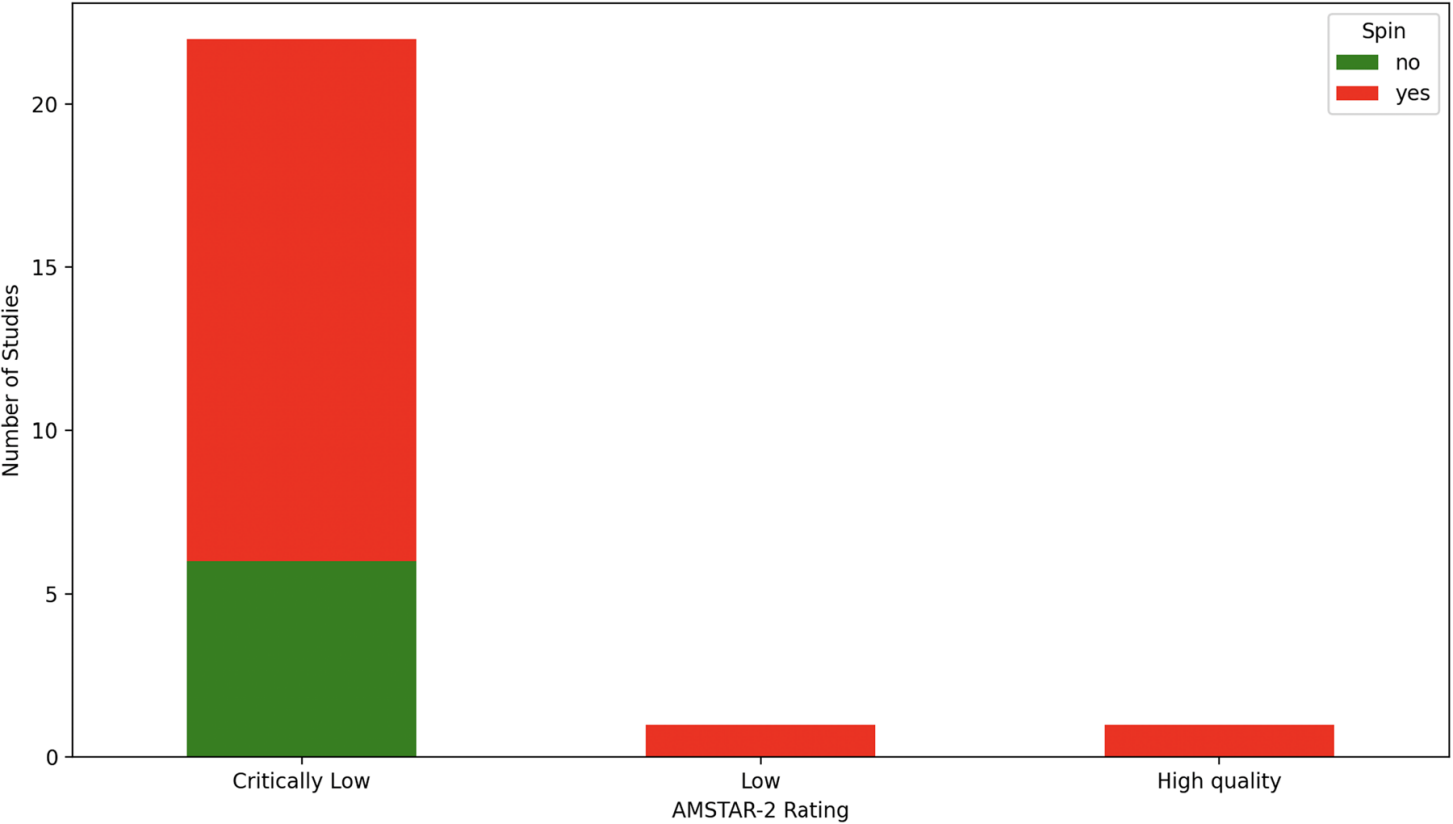
Distribution of studies by spin presence according to AMSTAR‐2 quality rating.

Inter‐reviewer agreement was substantial across the AMSTAR‐2 ratings (*κ* = 0.839; standard error = 0.1576; 95% confidence interval = 0.532–0.999) (Table 3). Following conflict resolution, 91.7% (22/24) of reviews were classified as critically low, 4.17% (1/24) as low and 4.17% (1/24) as high. No moderate AMSTAR‐2 scores were obtained. The distribution of studies by spin presence according to AMSTAR‐2 quality rating is shown in Figure 2.

**Table 3:** AMSTAR‐2 ratings identified by reviewer 1, reviewer 2 and post‐conflict resolution.

AMSTAR‐2 rating	Percentage of reviews with AMSTAR‐rating, reviewer 1	Percentage of reviews with AMSTAR‐rating, reviewer 2	Percentage of reviews with AMSTAR‐rating, post‐conflict resolution
Critically low	87.5% (21/24)	83.3% (20/24)	91.7% (22/24)
Low	8.33% (2/24)	12.5% (3/24)	4.17% (1/24)
High	4.17% (1/24)	4.17% (1/24)	4.17% (1/24)

### Assessment of spin

**Figure 3:**
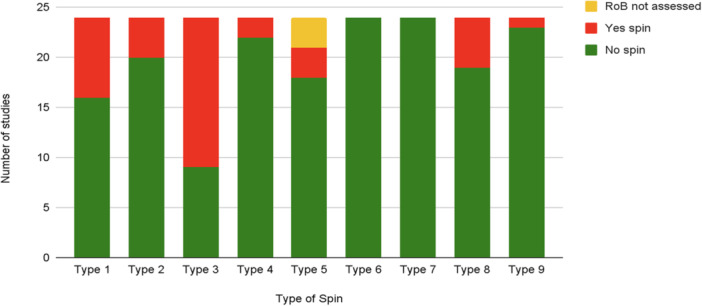
Number of reviews containing each type of spin.

After consensus, 75% (18/24) of abstracts were identified to contain at least one form of spin. A total of 37.5% (9/24) of reviews contained one type of spin, 12.5% (3/24) contained two types and 25.0% (6/24) contained three or more types of spin. The most prevalent type of spin was Type 3, ‘Selective reporting of or overemphasis on efficacy outcomes or analysis favoring the beneficial effect of the experimental intervention’ observed in 62.5% (15/24) of reviews (Table 1 and Figure 3). Other common types included Type 1, ‘Conclusion contains recommendations for clinical practice not supported by the findings’, present in 33.3% (9/24) of reviews, and Type 8, ‘Conclusion extrapolates the review's findings from a surrogate marker or a specific outcome to the global improvement of the disease’, present in 20.8% (5/24) of reviews. Three reviews did not assess RoB and, therefore, could not be evaluated for Type 5 spin (‘Conclusion claims the beneficial effect of the experimental treatment despite high risk of bias in the primary studies’) [12, 31, 47]. As a result, calculations for Type 5 spin were based on 21 reviews. Types 6 and 8 spin were absent from all abstracts reviewed. Table S2 outlines the comprehensive breakdown of the spin types identified.

**Figure 4:**
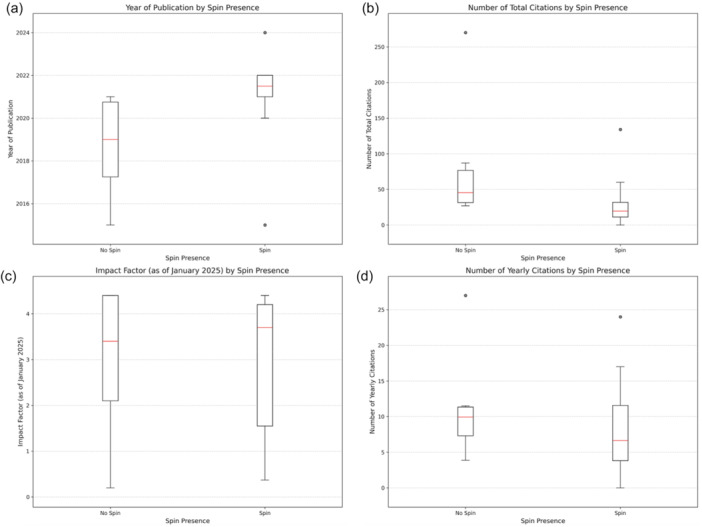
Box‐plots comparing median (interquartile range) values for (a) year of publication, (b) number of total citations, (c) number of yearly citations and (d) impact factor, for included reviews with no spin versus spin.

Reviews with at least one form of spin had a significantly later median year of publication compared to those with no spin (*p* = 0.011), with a median year of 2021.5 (IQR: 1 year) in the spin group versus 2019 (IQR: 3.5 years) in the no‐spin group. The number of total citations per review was significantly greater in the no‐spin group compared to the spin group (*p* = 0.021), with a median of 19.5 (IQR: 7.05 citations) in the spin group compared to 45.5 (IQR: 27.1 citations) in the no‐spin group. There was no significant difference in the average number of yearly citations in the no‐spin group compared to the spin group (*p* = n.s), with medians of 9.4 (IQR: 4.0) and 6.6 (IQR: 7.8), respectively. There was no significant difference in journal impact factor between groups (*p* = n.s), with medians of 3.7 (IQR: 2.7) and 3.4 (IQR: 2.3), in the spin and no‐spin groups, respectively (Figure 4a–d).

**Figure 5:**
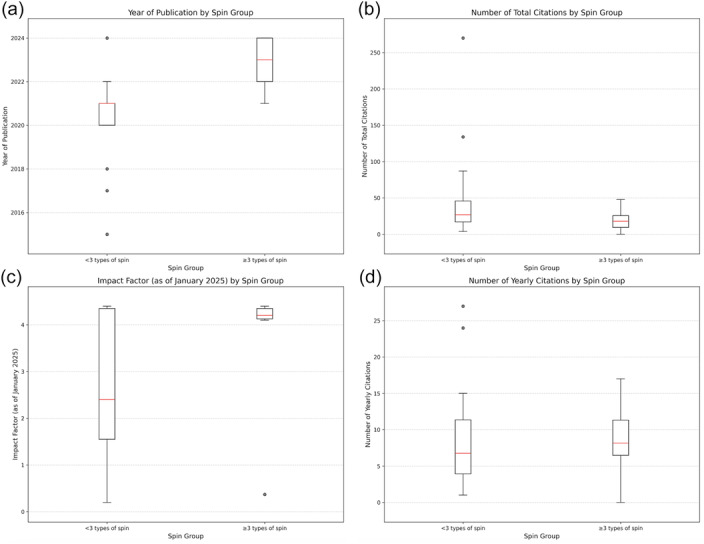
Box‐plots comparing median (interquartile range) values for (a) year of publication, (b) number of total citations, (c) number of yearly citations and (d) impact factor, for included reviews with less than three and greater than or equal to three types of spin.

Reviews with three or more types of spin had a significantly later median year of publication than those with fewer than three types (*p* = 0.007), with median years of 2023 (IQR: 2 years) in the group with three or more types of spin and 2021 (IQR: 1 year) in the group with fewer than three types of spin. There were no significant differences in total citations between groups (*p* = n.s), with medians of 18 (IQR: 16.3 citations) and 27 (IQR: 28.8 citations) in the three or more and the fewer than three types of spin groups, respectively. There was no significant difference in the average number of yearly citations in the three or more and the fewer than three types of spin groups (*p* = n.s), with medians of 6.8 (IQR: 7.4) and 8.2 (4.9), respectively. There were no statistically significant differences in impact factor between these groups (*p* = n.s), with medians of 4.2 (IQR: 0.2) and 2.4 (IQR: 2.8) in the three or more and less than three types of spin groups, respectively (Figure 5a–d).

## DISCUSSION

The primary conclusions of this study are threefold. First, the majority (75%) of published SR and MA comparing ACLR with or without LET or ALLR exhibit spin, suggesting that most SR and MAs may be overselling their findings in the abstract without adequate supporting data, which is especially detrimental for clinicians who rely on abstracts as a synopsis for decision making. Second, reviews with a higher degree of spin have a significantly more recent median year of publication, raising concern that newer literature may be more prone to overstating benefits, potentially distorting current clinical practice trends. Third, most reviews (91.7%) included have a critically low rating as per AMSTAR‐2 criteria, highlighting a need for caution when integrating these findings into patient care should they be based on suboptimal methodological quality.

The common presence of spin published in the abstracts of SRs and MAs in orthopaedics is a known phenomenon. SR and MAs examining spin in ACL ligament reconstruction [19], rotator cuff tear repair [3], proximal humeral fractures [25], Achilles tendon rupture [10], hip dysplasias [48] and osteoarthritis [46] have identified spin in of the abstracts of 34.2%–83% of their included reviews. Interestingly, Type 3 spin (‘Selective reporting of or overemphasis on efficacy outcomes or analysis favoring the beneficial effect of the experimental intervention’) was the most prevalent type of spin in our study, which has also been the case in other orthopaedic reviews [10, 25]. An example of Type 3 spin from this systematic search is observed in a review with a purpose stated as ‘[comparing] subjective outcomes, knee stability, and failure rates’ in ACL revision with and without a lateral extra‐articular procedure [18]. The abstract highlighted significant reductions in failure rate and postoperative pivot shift with the combined procedure. However, the review abstract did not report non‐significant findings in subjective outcomes in the abstract, including the Lysholm, International Knee Documentation Committee (IKDC) and Tegner scores, despite the evaluation of said outcomes being part of the stated purpose. This selective reporting overemphasizes the intervention's beneficial effects by highlighting only positive results, potentially misrepresenting the overall findings and leading to clinical decision‐making that may adversely impact patient care (no significant difference in patient‐reported outcomes). Similarly, type 3 spin was observed in a review assessing patient function, graft stability and rupture rates after ACLR with and without ALLR [9]. The abstract only reported higher graft rupture rates in isolated ACL reconstruction, omitting data on graft stability and functional outcomes, both primary objectives. Regardless of statistical significance, excluding these findings may mislead readers and neglect important information critical for patient care.

The prevalence of spin identified in this study highlights the need to critically appraise SRs and MAs to ensure clinical recommendations are based on transparent reporting rather than selective emphasis on favourable outcomes. In orthopaedics, the wide range of outcome measures, such as return to sport, pivot shift and patient‐reported scores like IKDC‐7 and knee osteoarthritis and outcome score, allows for flexible interpretation, increasing the risk of biased reporting. Spin in SR and MA abstracts is especially relevant, as these summaries often guide clinical decision‐making in evidence‐based practice [26]. This is particularly important for evolving procedures like LET and ALLR. Given that many clinicians rely on abstracts, the presence of bias at this level can significantly affect interpretation and patient care [8]. Ultimately, if the decisions made by clinicians are based on abstracts that inherently contain information that is not supported by evidence, patients may not only be receiving suboptimal care, but even unnecessary harm from these interventions.

Interestingly, this review identifies that recent reviews in ACLR with anterolateral procedures show a greater degree of spin. Reviews with spin and those with three or more types of spin had significantly later median publication years (*p* = 0.007). A recent study assessing spin in SR and MA related to treating proximal humerus fractures had a similar finding, where abstract spin was significantly more likely to appear in recent publications (odds ratio = 1.25; 95% confidence interval = 1.02–1.52) [25]. This trend may partially reflect the growing volume of literature in recent years [6], with most reviews in this analysis published in the last five years, potentially increasing pressure for authors to have their work ‘stand out’.

Beyond publication volume, academic and professional pressures may contribute as well. In a North American context, there is a rising demand for research productivity in applications for residency and advanced training, described as an ‘arms race’, with applicants facing intense competition to enhance their credentials through publications [14, 20, 45]. This competitive environment may incentivize authors, intentionally or unintentionally, to frame findings more favourably for publication. While no single factor may be directly responsible for the increasing presence of spin in abstracts in recent years, it is important to recognize the multifactorial and systemic pressures that may impact spin in publication.

Of note, several reviews included in this paper were rated as a ‘critically low quality’ as per the AMSTAR‐2 criteria. This is not a novel finding, as previous research has consistently demonstrated that the methodological quality of SRs and MAs, as assessed by AMSTAR‐2, tends to fall within the low or critically low range [3, 10, 19, 28, 46, 48]. The use of critical appraisal tools such as AMSTAR‐2 is crucial not only for identifying reviews with high methodological quality but also for guiding researchers toward contributing more rigorous reviews to the literature. However, from a clinical perspective, recognizing that most available evidence lies in SR and MAs which fall below acceptable methodological quality standards raises the concern that the overall standard quality for publication of SRs and MAs currently available is insufficient to meet the desired standards as per the AMSTAR‐2 criteria. Low AMSTAR‐2 scores reflected methodological shortcomings, with one notable phenomena being the inappropriate use and lack of discussion of the impact of pooled data from level III and level IV evidence (AMSTAR‐2 question 14), resulting in high heterogeneity and thus potentially misleading conclusions. Literature exists that promotes the idea that MA should ideally focus on homogeneous, high‐quality reviews (Levels I or II) to minimize confounding bias and enhance the reliability of conclusions, thus leading to more accurate insight for clinical practice [21]. The practice of pooling data is often employed to achieve seemingly robust results, driven by the limited availability of high‐level evidence. Indeed, this is expected in a field with relatively new and inconclusive findings, such as ACLR with and without LET or ALLR. However, this must be acknowledged and discussed in the publication; otherwise, this pursuit of statistical significance may paradoxically reduce the meaningfulness of findings if excessive heterogeneity compromises their reliability. Improving the quality of SRs and MAs requires stricter adherence to AMSTAR‐2 criteria, including comprehensive literature searches, clear inclusion criteria and rigorous RoB assessments. Standardizing methodologies and outcome measures across reviews can enhance comparability and clinical relevance while also accounting for population‐specific factors to help avoid misleading generalizations. Incorporating these practices and established reporting guidelines would strengthen the reliability and applicability of SRs and MAs [38]. Journals could further support quality improvement by requiring standardized methodological assessments with a threshold for publication and formal spin evaluations before publication [27, 38, 39].

Aside from contributing to the ongoing critical appraisal of research, particularly reviews that directly impact patient care, this study has several notable strengths. First, the standardized methodology ensured a comprehensive assessment of both spin and methodological quality using the AMSTAR‐2 tool. The use of duplicate data extraction and independent rating, with conflict resolution by a senior reviewer, minimized the potential for subjective bias and improved the reliability of our findings. Additionally, including reviews evaluating ACLR with and without ALLR or LET without restrictions on the year of publication allowed for a broad analysis. Given that this is a relatively new and underexplored area in orthopaedic research, this approach offers valuable insights into the current state of the literature and evolving trends over time.

Despite its strengths, this research study has several limitations. The identification of spin, although based on a standardized classification system [51], remains inherently subjective. Similarly, the AMSTAR‐2 criteria, while designed to provide a standardized assessment of methodological quality, are also subject to interpretation. Another limitation was that the spin assessment was based solely on the information provided within each review and therefore excluded studies from the Type 5 spin assessment if they did not assess RoB. While omitting the RoB assessment is not standard practice in SRs, it would also be inappropriate to infer spin in the absence of an explicit quality appraisal. Additionally, although many studies cite the PRISMA guidelines, actual adherence may not always be rigorous. As our data collection focused solely on whether PRISMA was cited, we did not evaluate adherence to the complete checklist; therefore, citation alone may overestimate methodological transparency and reporting quality. While this study provides insight into trends of spin within this context, its cross‐sectional design limits the ability to establish causality, and its generalizability to other orthopaedic or medical reviews remains unknown. Caution should be exercised when extrapolating these findings to other fields within medicine. Furthermore, the focus on abstracts may overlook additional instances of spin that could be present in full‐text articles. To further the quality of information in abstracts and, ultimately, the evidence from which clinicians draw for patient care, future reviews should incorporate formal spin assessment as a standard component of critical appraisal.

## CONCLUSION

Spin is highly prevalent in SR and MA evaluating ACLR with and without LET or ALLR, particularly in recent publications. Given that many reviews exhibit critically low methodological quality, efforts should focus on strengthening adherence to reporting standards and reducing spin to ensure the publication of unbiased evidence that informs clinical practice.

## AUTHOR CONTRIBUTIONS

*Screening, extraction and writing*: Adela Gottardi and Kieran Chalmers. *Screening, extraction, writing, editing and idea conception*: Prushoth Vivekanantha. *Writing and editing*: Jeffrey Kay and Mikael Sansone. *Writing, editing and supervision*: Darren de SA.

## CONFLICT OF INTEREST STATEMENT

The authors declare no conflicts of interest.

## ETHICS STATEMENT

There are no relevant ethical disclosures pertaining to research involving human participants and/or animals, and informed consent was not necessary to develop this manuscript.

## Supporting information

Supporting Data no highlights.

## Data Availability

Data may be made available upon reasonable request at prushoth.vivekanantha@medportal.ca.
