# Supplementary material for: Three in every four systematic reviews and meta‐analyses on concomitant anterior cruciate ligament reconstruction and anterolateral complex procedures present at least one type of spin in the abstract
Source: Knee Surg Sports Traumatol Arthrosc. 2025 Jul 13;34(6):1986–99. doi: 10.1002/ksa.12787 (PMC13266964; doi:10.1002/ksa.12787)
Supplement: Supplementary file 1 — Supporting Data no highlights. [file KSA-34-1986-s001.docx]

**SUPPLEMENTARY DATA**

**Supplementary Digital Material Table 1: Search Criteria used in PubMed, EMBASE, and MEDLINE[Ovid]**

|  | Search on PubMed, EMBASE, AND MEDLINE[Ovid] |
| --- | --- |
| 1. | Lateral extra-articular tenodesis OR LET |
| 2. | Anterolateral ligament OR ALL |
| 3. | Anterolateral complex |
| 4. | Anterior cruciate ligament OR ACL |
| 5. | (1 OR 2 OR 3) and 4 |

**Supplementary Digital Material Table 2: Comprehensive Breakdown of Conflict-Resolved Spin Types Across Reviews and Corresponding AMSTAR-2 Ratings**

| **Review** | **Type 1** | **Type 2** | **Type 3** | **Type 4** | **Type 5** | **Type 6** | **Type 7** | **Type 8** | **Type 9** | **AMSTAR-2** |
| --- | --- | --- | --- | --- | --- | --- | --- | --- | --- | --- |
| **Agarwal [1]** | no | no | yes | no | no | no | no | no | no | Critically Low |
| **Hurley [24]** | yes | no | no | no | no | no | no | no | no | Critically Low |
| **Onggo [36]** | no | no | yes | no | no | no | no | no | no | Critically Low |
| **Xu [51]** | no | no | yes | no | no | no | no | no | no | Critically Low |
| **Na [35]** | yes | no | yes | no | no | no | no | no | no | Critically Low |
| **Damayanthi [11]** | yes | no | yes | no | no | no | no | yes | yes | Critically Low |
| **Bosco [7]** | yes | yes | yes | no | no | no | no | yes | no | High quality |
| **Hewison [23]** | no | no | no | no | no | no | no | no | no | Critically Low |
| **Boksh [5]** | no | no | yes | no | yes | no | no | no | no | Critically Low |
| **Ariel de Lima [2]** | yes | no | yes | no | no | no | no | yes | no | Critically Low |
| **Mao [33]** | no | no | no | no | yes | no | no | no | no | Critically Low |
| **Park [37]** | yes | yes | yes | no | no | no | no | yes | no | Low |
| **Kunze [31]** | no | no | no | no | no | no | no | no | no | Critically Low |
| **Yin [53]** | no | no | no | no | no | no | no | yes | no | Critically Low |
| **Saithna [43]** | yes | yes | yes | no | yes | no | no | no | no | Critically Low |
| **Bucar [9]** | no | no | yes | no | no | no | no | no | no | Critically Low |
| **Song [48]** | no | no | yes | no | RoB not assessed | no | no | no | no | Critically Low |
| **Delaloye [12]** | no | no | no | no | RoB not assessed | no | no | no | no | Critically Low |
| **Feng [15]** | no | no | yes | no | no | no | no | no | no | Critically Low |
| **Beckers [4]** | no | no | no | no | no | no | no | no | no | Critically Low |
| **Devitt [13]** | no | no | no | no | no | no | no | no | no | Critically Low |
| **Grassi [18]** | yes | yes | yes | yes | no | no | no | no | no | Critically Low |
| **Littlefield [32]** | no | no | no | no | RoB not assessed | no | no | no | no | Critically Low |
| **Rhatomy [41]** | no | no | yes | yes | no | no | no | no | no | Critically Low |
